# Association of the Triglyceride‐Glucose Index With Stroke and All‐Cause Mortality in Patients With Coronary Artery Disease: A Nationwide Cohort Study From 1999 to 2018

**DOI:** 10.1002/mco2.70422

**Published:** 2025-10-09

**Authors:** Li‐Xin Huang, Tao Sun, Jun Sun, Zhi‐Min Wu, Yi‐Bo Zhao, Ming‐Yang Li, Qing‐Yi Huo, Bao‐Yu Zhang, Cong Ling, Chuan Chen, Hui Wang

**Affiliations:** ^1^ Department of Neurosurgery The Third Affiliated Hospital of Sun Yat‐Sen University Guangzhou China

**Keywords:** ACM, CAD, NHANES, stroke, TyG index

## Abstract

The triglyceride‐glucose (TyG) index is related to various cardiovascular diseases, but its relationship with stroke and all‐cause mortality (ACM) in individuals with coronary artery disease (CAD) is still not well understood. This research sought to analyze the interaction between the TyG index and the occurrence of stroke and ACM in CAD participants. The dataset was derived from the National Health and Nutrition Examination Survey (NHANES), with 809 CAD patients included from 1999 to 2018. TyG index was determined by ln[fasting triglycerides (mg/dL) × fasting glucose (mg/dL)/2]. Findings showed that heightened TyG index values were markedly associated with a greater risk of stroke; a U‐shaped interconnection was detected between the TyG index and stroke risk, with the threshold at 8.14. Individuals with a TyG index exceeding this threshold exhibited a markedly higher rate of stroke occurrence. Additionally, a J‐shaped correlation was observed between the TyG index and ACM, with the threshold at 9.25, above which the risk of death increased. These findings indicate that the TyG index could act as a practical indicator for predicting stroke and ACM among CAD patients, particularly when considering threshold values.

## Graphical abstract

This study investigates the association between the triglyceride‐glucose (TyG) index and the risk of stroke and all‐cause mortality in patients with coronary artery disease (CAD). Analysis of data from 809 CAD patients in the NHANES (1999‐2018) revealed a U‐shaped relationship between the TyG index and stroke risk, with a threshold of 8.14. A J‐shaped relationship was observed between the TyG index and all‐cause mortality, with a threshold of 9.25. Higher TyG values are associated with an increased risk of both stroke and death, highlighting the TyG index as a potential predictor in CAD patients.

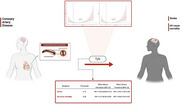

## 1 Introduction

Cardiovascular diseases (CVDs), notably coronary artery disease (CAD) and stroke, continue to be the primary origins of global mortality [1, 2]. As noted by the Global Burden of Disease 2021 investigation, stroke remains a significant global driver of mortality. There were approximately 6.55 million deaths due to stroke worldwide in 2021, with the age‐standardized mortality stood at 43.5 cases for every 100,000 person‐years [1]. The occurrence rate of stroke has been steadily increasing, underscoring the need for effective biomarkers to identify individuals at risk and intervene early [3].

One key factor contributing to cardiovascular events and poor prognosis is insulin resistance (IR) [4]. IR bears a close correlation with hyperglycemia, dyslipidemia, and hypertension, and is particularly prevalent in patients with CAD [5]. IR not only accelerates disease progression but also significantly increases the risk of stroke [5, 6]. However, current methods for evaluating IR, such as the intravenous glucose tolerance test and the hyperinsulinemic‐euglycemic clamp, are complex, expensive, and not suitable for large‐scale epidemiological studies [7].

The triglyceride‐glucose (TyG) index, integrating fasting triglyceride (TG) and glucose concentrations, provides a simpler and more economically feasible option for assessing IR [8]. Prior inquiry has shown that the TyG index is strongly linked to the risks of cardiovascular and metabolic conditions [9]. Nonetheless, its function in forecasting stroke, all‐cause mortality (ACM), and cardiovascular mortality in CAD populations has been insufficiently probed.

Leveraging information from the National Health and Nutrition Examination Survey (NHANES) carried out from 1999 to 2018, the research intends to assess the TyG index's value in predicting stroke and mortality risks among CAD patients, thus providing new insights for clinical risk stratification.

## 2 Results

### 2.1 Baseline Traits by Stroke Status

**FIGURE 1:**
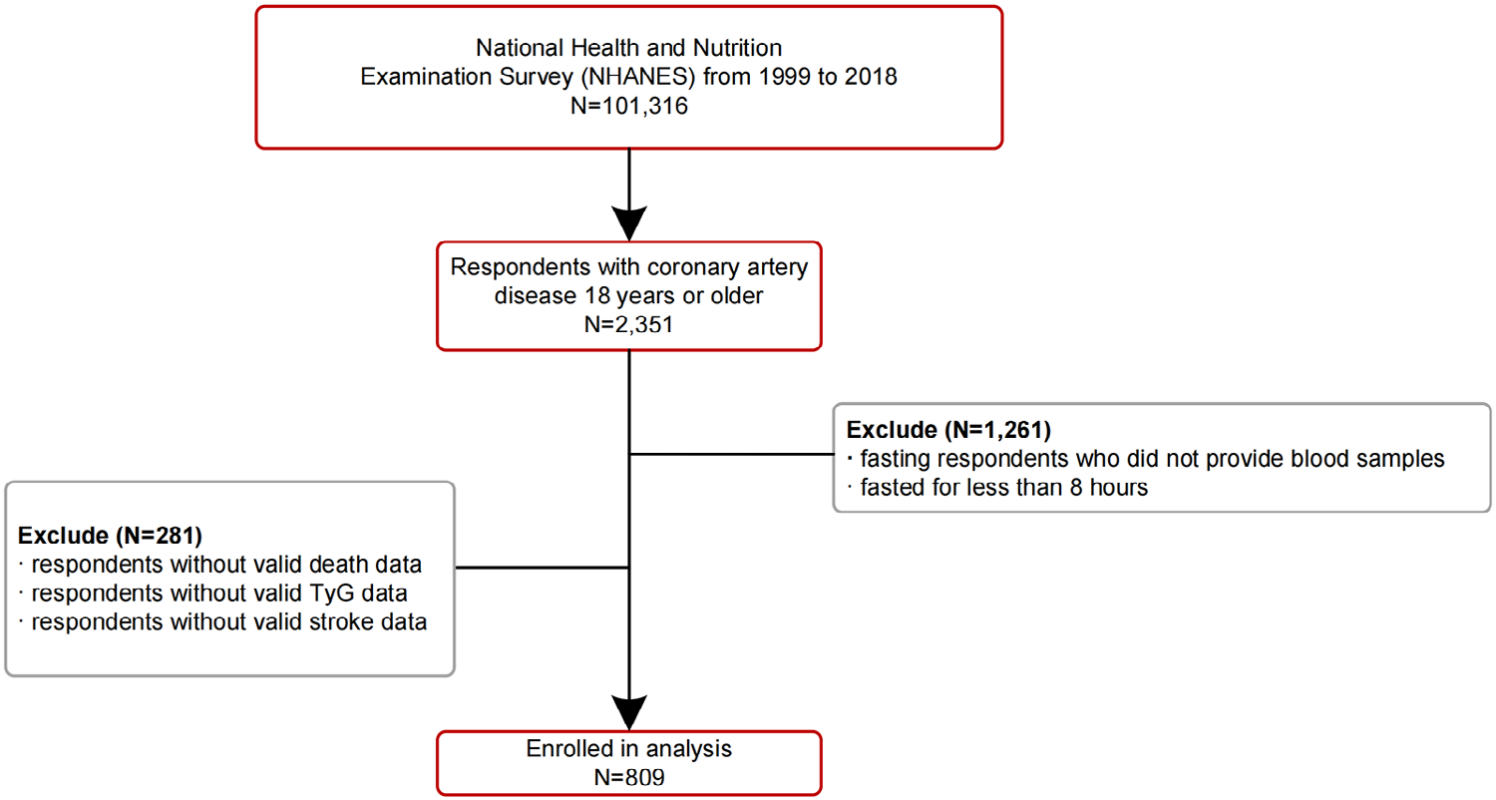
Screening flow of respondents.

**FIGURE 2:**
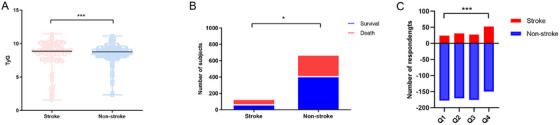
Analysis of triglyceride‐glucose (TyG) index in stroke and non‐stroke subjects. (A) Comparison of TyG index between stroke and non‐stroke groups (***p < 0.001). (B) Comparison of all‐cause mortality between stroke and non‐stroke groups (*p < 0.05). (C) Association of TyG index quartiles with stroke prevalence (***p < 0.001). The TyG index was categorized into quartiles (Q1–Q4).

This study included a total of 809 participants (Figure 1). According to the participants' responses, they were split into two groups: stroke and non‐stroke (Table 1). Stroke patients showed a higher proportion of males, heart failure history, and exhibited lower levels of education compared to non‐stroke respondents (all *p* < 0.05). Additionally, stroke cases displayed elevated levels of fasting blood glucose (FBG), total cholesterol (TC), TG, and TyG index (*p* < 0.05) (Figure 2A). Stroke respondents also exhibited a notably greater ACM rate (50.7% vs. 39.1%, *p* = 0.012; Figure 2B), but no statistically significant disparity was found in cardiovascular mortality rates (3.7% vs. 2.4%, *p* = 0.696).

**TABLE 1:** Baseline characteristics of the study participants grouped by stroke status.

	Total (*n* = 809)	Stroke (*n* = 134)	Non‐stroke (*n* = 675)	*p* value
Age, years, mean (SD)	68.4 ± 11.1	70.1 ± 10.5	68.1 ± 11.2	0.055
Gender, *n* (%)				0.020
Male	552 (68.2)	80 (59.7)	472 (69.9)	
Female	257 (31.8)	54 (40.3)	203 (30.1)	
BMI, kg/m^2^, mean (SD)	29.4 ± 6.0	29.5 ± 5.6	29.4 ± 6.1	0.671
Waist circumference, cm, mean (SD)	104.9 ± 14.9	103.8 ± 14.0	105.2 ± 15.1	0.363
Race, *n* (%)				0.689
Hispanic	141 (17.4)	26 (19.4)	115 (17.0)	
NH White	525 (64.9)	83 (61.9)	442 (65.5)	
NH Black	92 (11.4)	18 (13.4)	74 (11.0)	
Multiracial/other	51 (6.3)	7 (5.3)	44 (6.5)	
PIR, mean (SD)	2.6 ± 1.6	2.5 ± 1.4	2.6 ± 1.6	0.703
Education level, *n* (%)				0.006
Below high school	266 (32.9)	59 (44.0)	207 (30.1)	
High school graduate or GED	169 (20.9)	28 (20.9)	141 (20.9)	
Some college or above	374 (46.8)	47 (35.1)	327 (49.0)	
Marital status, *n* (%)				0.126
Married or living with a partner	534 (66.0)	79 (59.0)	455 (67.4)	
Never married	30 (3.7)	5 (3.7)	25 (3.7)	
Widowed, divorced, or separated	242 (29.9)	50 (37.3)	192 (28.4)	
Nicotine exposure, *n* (%)				0.595
Never	132 (16.3)	18 (13.4)	114 (16.9)	
Former	13 (1.6)	2 (1.5)	11 (1.6)	
Now	650 (80.3)	112 (83.4)	538 (79.7)	
Alcohol use, *n* (%)				0.052
Non‐drinker	253 (31.3)	54 (40.3)	199 (29.5)	
1–5 drinks/month	411 (50.8)	53 (39.6)	358 (53.0)	
6–10 drinks/month	3 (0.4)	1 (0.7)	2 (0.3)	
>10 drinks/month	3 (0.4)	1 (0.7)	2 (0.3)	
Medical history, *n* (%)				
Hypertension	587 (72.6)	104 (77.6)	483 (71.6)	0.232
Diabetes	338 (41.8)	64 (47.8)	274 (40.6)	0.124
Heart failure	231 (28.6)	58 (43.3)	173 (25.6)	< 0.001
Angina/angina pectoris	266 (32.9)	50 (37.3)	216 (32.0)	0.407
Cancer	173 (21.4)	31 (23.1)	142 (21.0)	0.589
Antihyperlipidemic	590 (72.9)	96 (71.6)	494 (73.2)	0.750
Antidiabetic	230 (28.4)	42 (31.3)	188 (27.9)	0.404
SBP, mmHg, mean (SD)	133.3 ± 22.4	134.0 ± 23.6	133.1 ± 22.2	0.496
DBP, mmHg, mean (SD)	67.2 ± 12.5	66.9 ± 13.1	67.3 ± 12.4	0.317
Laboratory measurements, mean (SD)				
HbA1c, %	6.24 ± 1.34	6.28 ± 1.44	6.24 ± 1.33	0.074
FBI, pmol/L	86.9 ± 69.1	104.5 ± 86.9	83.6 ± 65.1	0.282
TC, mmol/L	4.51 ± 1.19	4.52 ± 1.41	4.51 ± 1.15	0.090
HDL‐C, mmol/L	1.27 ± 0.39	1.30 ± 0.43	1.26 ± 0.38	0.065
LDL‐C, mmol/L	2.50 ± 0.97	2.53 ± 1.16	2.49 ± 0.93	0.094
ALB, g/L	41.5 ± 3.6	40.4 ± 4.0	41.7 ± 3.5	0.099
ALT, IU/L	23.5 ± 14.0	21.9 ± 12.6	23.8 ± 14.3	0.385
AST, IU/L	25.3 ± 10.4	24.6 ± 9.1	25.5 ± 10.6	0.394
BUN, mmol/L	6.68 ± 3.42	7.17 ± 3.76	6.58 ± 3.35	0.087
GGT, IU/L	34.9 ± 42.3	33.4 ± 41.2	35.2 ± 42.6	0.701
LDH, IU/L	147.4 ± 38.1	153.0 ± 41.6	146.3 ± 37.3	0.222
TBIL, µmol/L	12.6 ± 5.7	12.1 ± 5.9	12.7 ± 5.6	0.655
SUA, IU/L	365.5 ± 96.3	366.9 ± 103.5	365.2 ± 94.9	0.056
SCR, µmol/L	100.9 ± 70.0	107.5 ± 57.8	99.6 ± 72.1	0.551
TG, mg/dL	140.9 ± 90.3	169.8 ± 121.0	135.2 ± 81.8	< 0.001
FBG, mg/dL	123.0 ± 46.3	135.6 ± 64.6	120.0 ± 41.4	< 0.001
TyG index, mean (SD)	8.86 ± 0.68	9.04 ± 0.89	8.83 ± 0.62	< 0.001
All‐cause mortality, *n* (%)	332 (41.0)	68 (50.7)	264 (39.1)	0.012
Cardiovascular mortality, *n* (%)	21 (2.6)	5 (3.7)	16 (2.4)	0.696

*Note*: Data presented as mean (standard deviation, SD) for continuous and numbers; (%) values for categorical. Chi‐squared test with Rao and Scott's second‐order correction; Wilcoxon rank‐sum test for complex survey samples.

Abbreviations: ALB, albumin; ALT, alanine aminotransferase; AST, aspartate aminotransferase; BMI, body mass index; BUN, blood urea nitrogen; DBP, diastolic blood pressure; FBG, fasting blood glucose; FBI, fasting blood insulin; GED, general educational development test; GGT, glutamyl transpeptidase; HbA1c, hemoglobin A1c; HDL, high‐density lipoprotein; LDH, lactic dehydrogenase; LDL‐C, low‐density lipoprotein cholesterol; PIR, poverty‐income ratio; SBP, systolic blood pressure; SCR, serum creatinine; SUA, serum uric acid; TBIL, total bilirubin; TC, total cholesterol; TG, triglyceride; TyG, index triglyceride‐glucose.

### 2.2 Nonlinear Connections of the TyG Index With Stroke and ACM

**FIGURE 3:**
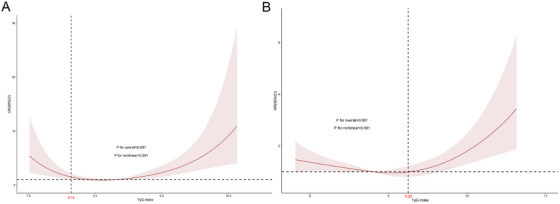
Multivariate adjusted spline curve of the association between the triglyceride‐glucose (TyG) and stroke (A) and between the TyG index and all‐cause mortality (B) in respondents with CAD. Odds ratios (OR) and hazard ratios (HR) were adjusted for age (as a continuous variable), gender, race, education level, marital status, BMI, nicotine exposure, and alcohol consumption. The solid line and red area represent the estimated values and their corresponding 95% CI. CI, confidence interval.

After dividing the TyG index into quartiles, a marked correlation with stroke was observed (*p* < 0.001) (Figure 2C). According to the TyG index quartiles outlined in Table 2, binary logistic regression results of the analysis indicated that both models 1 and 2 suggested a tendency for stroke incidence to rise as the TyG index elevated (*p* < 0.05). In Model 3 (adjusted for age, body mass index (BMI), gender, marital status, race, education level, nicotine exposure, and alcohol consumption), the odds ratio (OR) for the Q1 group was 1.00 (serving as the reference), while the ORs for the Q2, Q3, and Q4 groups were 1.19 (95% CI: 0.63–2.24), 1.05 (95% CI: 0.54–1.99), and 2.66 (95% CI: 1.47–4.76), respectively—all indicating a significant association with stroke incidence (*p* < 0.05). Within the continuous model, after adjusting for confounders, every 1‐unit elevation in the TyG index was linked to a 1.47‐times higher stroke incidence (OR: 1.47, 95% confidence interval [CI]: 1.04–2.08, *p* = 0.027) (Table 3). Further analysis using restricted cubic spline (RCS) showed a nonlinear dose–response correlation between the TyG index and stroke occurrence, featuring a significant U‐shaped interrelation (*p* < 0.001) (Figure 3A). The threshold point was 8.14 (likelihood ratio *p* < 0.001): beneath this threshold, every 1‐unit rise in the TyG index was associated with a 98% decrease in stroke risk (OR: 0.02, 95% CI: 0.003–0.07); beyond the threshold, the stroke risk went up by 193% (OR: 2.93, 95% CI: 1.96–4.39) (both *p* < 0.001) (Table 3).

**TABLE 2:** Associations between the TyG index and stroke and all‐cause mortality in patients with coronary heart disease.

	Quartiles of the TyG index				
	Q1 (6.85–8.41)	Q2 (8.42–8.81)	Q3 (8.82–9.27)	Q4 (9.28–11.46)	*p* value
Stroke (OR (95% CI))					
Number of people	24	31	27	52	−
Model 1	Ref.	1.34 (0.76–2.38)	1.13 (0.63–2.05)	2.57 (1.51–4.37)	< 0.001
Model 2	Ref.	1.31 (0.74–2.33)	1.15 (0.64–2.08)	2.60 (1.53–4.44)	< 0.001
Model 3	Ref.	1.19 (0.63–2.24)	1.05 (0.54–1.99)	2.66 (1.47–4.76)	< 0.001
All‐cause mortality (HR (95% CI))					
Number of people	80	89	73	90	−
Model 1	Ref.	1.0 (0.74–1.35)	0.69 (0.50–0.94)	1.00 (0.74–1.35)	0.059
Model 2	Ref.	0.87 (0.64–1.17)	0.70 (0.51–0.97)	1.11 (1.06–1.09)	0.024
Model 3	Ref.	0.83 (0.58–1.18)	0.71 (0.49–1.03)	1.19 (1.02–1.72)	0.033

The TyG index was categorized into quartiles (Q1–Q4). Binary logistic regression models were used to estimate OR and 95% CI. Cox proportional hazard models were used to estimate HR and 95% CI. Model 1 was unadjusted; Model 2 was adjusted for age, race, and gender; and Model 3 was adjusted for age, gender, race, education level, marital status, body mass index, nicotine exposure, and alcohol use.

Abbreviations: CI, confidence interval; HR, hazard ratio; OR, odds ratio; Ref., reference; TyG, triglyceride‐glucose.

**TABLE 3:** Threshold effect analysis of TyG index on stroke and all‐cause mortality.^a^

Stroke	OR (95% CI)	*p* value
TyG index		
Fitting by the standard binary logistic regression model	1.47 (1.04–2.08)	0.027
Fitting by a two‐piecewise logistic regression model		
Inflection point	8.14	
TyG index < 8.14	0.02 (0.003–0.07)	< 0.001
TyG index ≥ 8.14	2.93 (1.96–4.39)	< 0.001
*p* for log‐likelihood ratio	< 0.001	

All‐cause mortality	HR (95% CI)	*p* value
TyG index		
Fitting by the standard Cox proportional risk model	1.13 (0.90–1.40)	0.291
Fitting by the two‐piecewise Cox proportional risk model		
Inflection point	9.25	
TyG index < 9.25	0.73 (0.53–0.99)	0.042
TyG index ≥ 9.25	2.58 (1.63–4.07)	< 0.001
*p* for log‐likelihood ratio	< 0.001	

*Note*: Binary logistic regression models were used to estimate OR and 95% CI. Cox proportional hazard models were used to estimate HR and 95% CI.

Abbreviations: CI, confidence interval; HR, hazard ratio; OR, odds ratio; TyG, triglyceride‐glucose.

^a^ Adjusted for age, gender, race, education level, marital status, body mass index, nicotine exposure, and alcohol use.

Moreover, Cox proportional hazards regression showed that 332 instances of ACM were documented during a median follow‐up duration of 103 months. Model 2 suggested that the TyG index showed a connection to an upward trend in ACM risk (*p* < 0.05). In model 3, adjusted for age, education level, gender, race, marital status, alcohol consumption, BMI, and nicotine exposure, the hazard ratio (HR) for the Q1 group was 1.00 (reference), while the HR among the Q2, Q3, and Q4 groups were 0.83 (0.58–1.18), 0.71 (0.49–1.03), and 1.19 (1.02–1.72), respectively, showing a significant association with ACM risk (*p* < 0.05) (Table 2). Within the continuous model, after adjusting for confounders, every 1‐unit elevation in the TyG index was linked to a 1.13‐times elevated risk of ACM (HR: 1.13, 95% CI: 0.90–1.40, *p* = 0.291) (Table 3). Additional analysis via RCS revealed a nonlinear dose–response correlation between the TyG index and ACM risk, characterized by a J‐shaped relationship (*p* < 0.001) (Figure 3B). The threshold point was 9.25 (likelihood ratio *p* < 0.001): beneath this threshold, a 1‐unit rise in the TyG index was linked to a 27% lower ACM risk (HR: 0.73, 95% CI: 0.53–0.99, *p* = 0.042); beyond this threshold, the ACM risk surged by 158% (HR: 2.58, 95% CI: 1.63–4.07, *p* < 0.001) (Table 3).

### 2.3 Heterogeneity in TyG Index Effects on Stroke and ACM Across Subgroups

**FIGURE 4:**
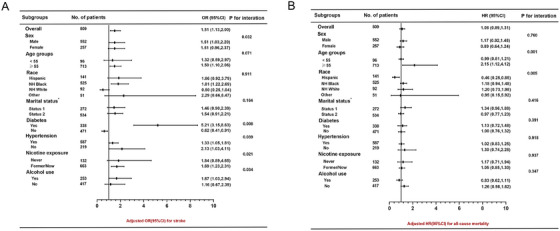
Stratified analysis of the association between the triglyceride‐glucose (TyG) and stroke (A), as well as between the TyG index and all‐cause mortality (B) in respondents with CAD. Stratified analyses of the associations between the TyG index, stroke, and all‐cause mortality among respondents with CAD. *Marital status: 1: married or living with a partner; 2: never married/widowed/divorced/separated. CI confidence interval, HR, hazard ratio; NH, non‐Hispanic; OR, odds ratio; TyG, triglyceride glucose.

To appraise the heterogeneity of the TyG index on stroke occurrence among CAD patients, we carried out a stratified assessment based on age, gender, marital status, race, diabetes, history of hypertension, alcohol consumption, and nicotine exposure (Figure 4A). Notable interaction effects were detected in subgroups with hypertension, nicotine exposure, diabetes, and alcohol consumption (all *p* < 0.05), whereas no meaningful interactions emerged in age, marital status, race, and gender. Among participants with diabetes, the TyG index had a significant bearing on stroke incidence (OR: 5.21, 95% CI: 3.15–8.63, *p* < 0.05). Among individuals with hypertension, the TyG index also showed a notable relevance to stroke incidence (OR: 1.33, 95% CI: 1.05–1.81, *p* < 0.05). Among those with nicotine exposure (OR: 1.69, 95% CI: 1.23–2.31, *p* < 0.05) and alcohol consumption subgroups (OR: 1.67, 95% CI: 1.03–2.94, *p* < 0.05), the TyG index revealed a link to the escalated incidence of stroke. Additionally, even though no significant interactions emerged in the age, race, gender, and marital status subgroups, a significant association was found among male participants, where the TyG index was linked to the occurrence of stroke (OR: 1.51, 95% CI: 1.03–2.20, *p* < 0.05). Among participants aged ≥ 55 years, the TyG index showed a striking connection with stroke incidence (OR: 1.50, 95% CI: 1.10–2.06, *p* < 0.05). Moreover, in the non‐Hispanic (NH) Black subgroup, the TyG index also showed a notable link to stroke incidence (OR: 1.81, 95% CI: 1.22–2.69, *p* < 0.05).

In a similar vein, to appraise the heterogeneity of the TyG index related to ACM risk in CAD patients, a stratified analysis was conducted on the basis of gender, race, age, marital status, history of hypertension, alcohol consumption, diabetes, and nicotine exposure (Figure 4B). Notable interaction effects were detected in age and race subgroups (both *p* < 0.05), whereas no notable interactions were identified in the remaining subgroups. Only in the ≥ 55 years subgroup, the TyG index showed a notable connection with ACM risk (HR: 2.15, 95% CI: 1.12–4.12, *p* < 0.05).

## 3 Discussion

Our results demonstrate a U‐shaped interrelation between the TyG index and stroke, featuring an inflection point determined at 8.14. This threshold was derived from the inflection point of the spline curve, which signifies a notable shift in how the TyG index correlates with the risk of stroke. While 8.14 is not the lowest point on the curve, it represents a clinically significant threshold at which stroke risk begins to rise. Similarly, a J‐shaped interrelation was identified between the TyG index and ACM among CAD patients. The process for determining this threshold involved *analyzing* the spline curve to identify key points, such as *local minima* and *maxima*, and selecting the value that most accurately reflects a clinically meaningful increase in stroke risk for the study population. Furthermore, stroke patients *exhibit higher ACM* in comparison to non‐stroke individuals, and we performed a Cox regression analysis with a corrected model, which indicated a notable statistical interrelation between the TyG index and ACM in CAD participants (*p* = 0.033). This research underscores the function of the TyG index in pinpointing high‐risk CAD groups susceptible to stroke and ACM, and it might provide insights for more focused approaches to prevention, screening, and clinical management.

IR has been linked to multiple clinical signs of metabolic syndrome, such as obesity, dyslipidemia, and hypertension [10]. Initially put forward in 2008, the TyG index is recognized as a dependable, economical, and straightforward substitute indicator for evaluating IR [8]. A wealth of clinical studies have probed the interrelation between the TyG index and CVD occurrence as well as mortality among both the distinct patient subsets and the general population [11, 12]. For instance, Yang X and colleagues demonstrated that a raised TyG index is related to poorer neurological outcomes, as well as a heightened risk of stroke occurrence and mortality [13]. Lee and colleagues suggested that the TyG index was competent to forecast short‐term functional outcomes in acute stroke survivors 3 months after symptom onset who received reperfusion intervention [14]. Zhao et al. examined its predictive value in diabetic acute ischemic stroke patients, finding the TyG index showed the most robust interrelation with cardiovascular event peril and proved suitable as a key indicator for risk stratifying in non‐ST elevation acute coronary syndrome cases (regardless of type 2 diabetes status) who underwent percutaneous coronary intervention [11]. While diabetes and hypertension are widely recognized as recognized risk determinants for stroke in prior literature, our preliminary analysis failed to identify notable differences in these factors between stroke and non‐stroke groups. However, stratified assessment demonstrated that the interaction between the TyG index and hypertension or diabetes had a significant impact on stroke incidence. Based on these findings, we recommend further research with larger sample sizes.

Among individuals with CAD, the TyG index might function as a useful outcome‐predictive indicator regarding adverse events that may occur later [15, 16]. Guerrero‐Romero and colleagues identified the TyG index as the most potent strategy for assessing IR, with notable predictive value, particularly in middle‐aged and younger individuals [17]. In addition, Navarro‐González et al. suggested that the TyG index may outperform TG and FBG as a screening tool for type 2 diabetes, highlighting that, in certain cases, it may provide a more accurate reflection of disease progression than individual glucose or TG levels [18]. Similarly, Sánchez‐Íñigo and colleagues documented a strong relationship between TyG index and cardiovascular adverse events, such as cerebrovascular conditions, coronary artery failure, and peripheral arterial disease, noting that it remains unaffected by confounding factors [19]. Therefore, these observations establish the TyG index serving as a trustworthy indicator with respect to various cardiovascular and metabolic conditions. The cumulative evidence underscores its capacity to forecast clinical outcomes in cerebrovascular and CVDs.

A report released by the World Stroke Organization (WSO) in 2022 revealed that from 1990 to 2019, global stroke occurrence has risen by 70%, while stroke‐related mortality rose by 43% [20]. In this study, patients with both stroke and CAD exhibited a notably higher ACM rate (*p* = 0.012). Ambrosy and colleagues carried out a cohort study on statin treatment and found that increased TG levels correlated with a greater likelihood of atherosclerotic cardiovascular events, yet a reduced probability of dying [21]. In a separate study, Lee and colleagues discovered a *J‐shaped* association between FBG concentrations and both ACM and cardiovascular events in elderly diabetic patients [22]. Zhang and colleagues examined the baseline TyG index and its relationship with the death rate among American adults with prediabetes or diabetes. Their findings showed a U‐shaped connection between the baseline TyG index and ACM in patients suffering from CVD, with a cutoff of 8.84 for CVD‐related death and 9.05 for ACM [12]. Furthermore, Yao and colleagues further observed that the TyG index‐mortality association varied by age: increased levels of the TyG index were associated with an escalated probability of ACM or non‐cardiovascular mortality only among individuals with type 2 diabetes who are under 65 years old, but not in older adults [23]. In contrast, our study shows a U‐shaped association between the TyG index and the stroke occurrence rate and a J‐shaped interrelation between the TyG index and the ACM among CAD patients.

Numerous investigations have laid the groundwork for a notable interrelation between the TyG index and the occurrence of atherosclerotic CVD, including stroke [24, 25]. A US cohort investigation revealed that heightened TyG index levels bore a relationship to an elevated stroke risk. Following adjustment for confounding variables, every single 1‐unit elevation in the TyG index correlated with a 32.1% greater stroke threat [25]. Correspondingly, another study found that each 1 standard deviation (SD) rise in the TyG index was linked to a 34% elevated stroke likelihood [26]. Shi et al. were the first to identify a standalone relationship between the TyG index and ischemic stroke among the general populace, highlighting its linear relationship. Their findings underscored the capacity of the TyG index to improve ischemic stroke risk grading [27]. A subsequent prospective large cohort enrolling individuals suffering from acute ischemic stroke revealed that the TyG index was tied to a greater risk of stroke recurrence and elevated 12‐month ACM [28]. More recently, Huang et al. investigated the TyG index trajectory among hypertensive patients and found that persistently high levels of the TyG index were associated with an elevated stroke risk, especially ischemic stroke [29]. Additionally, Wang and colleagues indicated that fluctuations in the TyG index could autonomously predict stroke occurrence [30]. Hong and colleagues also noted that a raised TyG index level serves as an independent forecaster of ischemic stroke [31]. A meta‐analysis encompassing eight cohort studies from China, Spain, South Korea, Argentina, and Iran observed that the TyG index autonomously correlates with stroke due to atherosclerosis‐related cerebrovascular events [24]. Furthermore, a 2022 meta‐analysis involving 11 studies reached a similar conclusion [32]. Cai et al. investigated the TyG index in severely sick individuals with ischemic stroke and ascertained that it is significantly correlated with higher mortality rates during hospitalization and in the intensive care unit (ICU) [33]. In 2024, Huo et al. showed that this index not only relates to stroke risk but also accounts for more than half of the link between stroke and BMI [34].

The exact mechanisms through which IR contributes to stroke remain undefined, but several potential pathways have been proposed: First, IR may lead to endothelial impairment, promoting the formation of foam cells and the progression of vulnerable plaques—vital stages in the advancement of atherosclerosis. Former investigations have indicated that the TyG index bears an interrelation with atherosclerosis and acts as a standalone predictor of plaque evolution [35, 36]. Additionally, IR, as a state of chronic mild inflammation, accelerates the evolution of atherosclerosis by increasing the formation of inflammatory markers [37]. Second, IR influences platelet activation, adhesion, and aggregation, potentially contributing to stroke via arterial narrowing or obstruction [38]. In the bargain, IR has been related to augmented activity of the sympathetic nerves and compromised autonomic cardiac function—both of which facilitate the development of acute heart and brain vascular episodes [21, 28, 39].

Our findings suggest that prioritizing TyG index screening in CAD patient management is warranted. Timely identification of stroke risk and possible unfavorable results may help slow the progression of the illness and alleviate socioeconomic pressures. The TyG index is both easily obtainable and budget‐friendly, yet research on its specific relationship with IR and stroke remains limited. Therefore, additional large‐scale prospective cohort studies and multi‐site trials that employ the TyG index as a predictive marker are crucial for improving its clinical management and utility in patients with stroke. Moreover, rigorously designed foundational inquiry is also required to investigate the underlying molecular pathways connecting the TyG index to stroke pathophysiology and its sequelae. While prior investigations have explored the TyG index–stroke association, this study further validates its clinical relevance and predictive value in CAD patients through long‐term, large‐scale cohort analysis. It also expands upon the clinical usefulness of the TyG index in stroke, establishing a basis for further research into the pathophysiological mechanisms underlying stroke in CAD patients. Notably, our findings differ from previous studies by revealing non‐linear interconnections between TyG and outcomes exclusively among CAD patients. While Yang et al. [13] and Shi et al. [27] reported linear correlations between TyG and stroke in general populations, our RCS analysis identified a U‐shaped curve for stroke (threshold: 8.14) and a J‐shaped curve for mortality (threshold: 9.25) in CAD patients. This suggests that TyG's predictive value is not monotonic in this high‐risk group—below the threshold, TyG may even correlate with reduced risk, which challenges the linear assumption in prior research [30]. These patterns imply that CAD patients may have distinct metabolic responses to TyG fluctuations, warranting tailored risk assessment. Furthermore, the J‐shaped connections between TyG and ACM in CAD patients, with a threshold of 9.25, are a novel finding. Unlike Zhang et al. [12], who reported a U‐shaped interaction of TyG‐mortality among general CVD patients (threshold value: 9.05), our study specifies that in CAD patients, mortality risk rises sharply only above 9.25, providing a more precise cutoff for identifying those needing aggressive risk reduction.

Merits of the research include its exclusive utilization of NHANES datasets, which follow standardized collection protocols to ensure reliability. We examined the dose‐dependent connection of the TyG index with stroke, pinpointing the threshold at which beneficial relationships occur. Nevertheless, it is imperative to take stock of certain constraints. To begin with, our study included participants from the United States, which may limit the global feasibility of our discoveries. Second, despite efforts to control for biases, the retrospective essence in this research could bring about remaining confounding. Third, the absence of real‐time surveillance of the TyG index impeded evaluating long‐duration trends. Furthermore, the primary analysis focused on the TyG index–stroke, lacking comparison to other non‐insulin‐based markers of IR. Fourth, CAD patients were categorized using self‐reported data rather than the latest diagnostic criteria, limiting sensitivity analyses to assess the robustness. The demographic characteristics and regional variability of the US‐only population may affect the generalizability. Thus, future studies could reproduce these results in multinational cohorts to enhance applicability. While prior research has explored the TyG index–stroke association among CAD patients, our research offers additional insights and new data points, providing a stronger foundation for future research and more precise clinical guidance. Finally, we recognize that these findings primarily apply to adult US populations with pre‐existing CAD. As such, the TyG index threshold requires further validation in diverse regional populations before broad generalization.

In conclusion, we uncovered significant interrelations of the TyG index with both stroke occurrence and ACM risk in patients with CAD. A U‐shaped interconnection was detected between the TyG index and stroke, the threshold value being 8.14, while a J‐shaped correlation was identified for ACM, featuring a threshold of 9.25. These findings indicate that the TyG index can act as a valuable biological marker aiding in high‐risk perception of CAD populations, holding promise for more targeted clinical intervention strategies.

## 4 Materials and Methodologies

### 4.1 Research Design and Study Participants

The dataset used in this research was sourced from NHANES, which employed a multi‐stage, stratified, cluster sampling design. It was carried out by the National Center for Health Statistics (NCHS), a component of the Centers for Disease Control and Prevention (CDC) [40]. The research protocol was granted approval by the NCHS Institutional Review Board, while all participants furnished written consent with full information. The NHANES database ensures the quality of data through standardized procedures, including laboratory quality control and data cleaning.

This analysis included adult patients diagnosed with CAD in the NHANES dataset from 1999 to 2018. CAD was identified as a self‐reported past history of coronary heart disease clinically diagnosed by a physician or other healthcare professional during in‐person interviews [41]. After excluding participants without blood samples or with fasting durations of less than 8 h (*n* = 1261), along with those lacking data on mortality, the TyG index, or stroke (*n* = 281), 809 patients with CAD were incorporated into the final analysis. Strict quality control procedures were implemented, including independent data entry by two researchers, cross‐checking, and final verification by the corresponding author.

### 4.2 TyG Index Calculation and Variable Definitions

The TyG index was computed with the following formula: ln[fasting TG (mg/dL) × fasting glucose (mg/dL)/2].

Data were derived from subjects who fasted for 8–24 h [42]. TG levels were quantified with Roche Hitachi 717/912, Modular P, and Cobas 6000 analyzers, while FBG was measured using Roche C501 (1999–2015) and C311 (2015–2018) devices. According to NHANES guidelines, no correction was necessary for results from different instruments.

Demographic characteristics, including race (Hispanic, NH White, NH Black, etc.), educational attainment (less than high school, high school graduate/GED, college or higher), marital status (cohabiting/married, unmarried, separated/widowed/divorced), and poverty‐income ratio (PIR), were collected through questionnaires. Smoking history (never, former, current), alcohol intake (no alcohol consumption, 1–5 drinks per month, 6–10 drinks per month, more than 10 drinks per month), and medical history of hypertension, heart failure, angina, and cancer were also recorded. Diabetes was characterized as satisfying two of the following circumstances: a history of diabetes reported by the participants themselves, fasting glucose levels of 126 mg/dL or higher, or the administration of antihyperglycemic medications.

Anthropometric measurements (e.g., blood pressure, weight, and waist circumference) were taken by mobile examination centers following standardized protocols. Laboratory tests covered lipid profiles, blood glucose, liver and kidney function, and other biomarkers (Table 1).

### 4.3 Outcome Events Definition

The primary outcomes included ACM and cardiovascular mortality. ACM was characterized as death from any cause, whereas cardiovascular mortality was determined using ICD‐10 codes (heart disease I00‐I51, cerebrovascular disease I60‐I69) [43, 44]. Mortality data were connected to the NCHS National Death Index up to December 31, 2019. The matching algorithm is described on the official website (https://www.cdc.gov/nchs/data‐linkage/mortality.htm). Stroke was defined according to participants' self‐reported confirmation of a physician‐diagnosed stroke [45].

### 4.4 Statistical Analysis

Sampling weights recommended by NHANES were utilized to adjust for the intricate survey design [46]. Means ± SDs were used to present continuous variables, while frequencies (with percentages) characterized categorical variables; the chi‐square test and Wilcoxon rank‐sum served to evaluate group differences.

For the purpose of evaluating the interrelations between the TyG index and outcome events, the index was categorized into quartiles (Q1–Q4). Multivariable Cox proportional‐hazards regression and binary logistic regression models were developed. Model 1 persisted unadjusted; Model 2 incorporated calibrations for race, age, and gender; and Model 3 added further calibrations for marital status, educational attainment, BMI, smoking, and alcohol intake. RCS analysis found application in investigating nonlinear interconnections, with thresholds determined via the maximum likelihood method. Missing data for continuous variables were handled with multiple imputations, and results were consistent with complete‐case analyses [47] (Table S1).

R 4.4.1 and SPSS 26.0 were the tools for all statistical computations, and statistical significance was determined by a two‐tailed *p*‐value < 0.05.

## Author Contributions

Hui Wang, Chuan Chen, and Cong Ling formulated the study concept and design. Li‐Xin Huang, Tao Sun, Jun Sun, and Zhi‐Min Wu were responsible for data collection and manuscript preparation. Li‐Xin Huang, Ming‐Yang Li, Yi‐Bo Zhao, and Qing‐Yi Huo contributed to the data analysis. Zhi‐Min Wu and Bao‐Yu Zhang participated in data interpretation. All contributors engaged in critical revisions of the manuscript, conducted reviews, gave their approval to the final draft, and took on accountability for the research. All authors have read and approved the final manuscript.

## Ethics Statement

The NCHS Institutional Review Board granted approval for this survey, and every participant submitted written consent with full information.

## Conflicts of Interest

The authors declare no conflicts of interest.

## Supporting information




**Supporting File 1**: mco270422‐sup‐0001‐SuppMat.docx.

## Data Availability

The data employed in this research are publicly available through the NHANES database; access can be obtained via the official NHANES website.
