# Supplementary material for: Association of the Triglyceride‐Glucose Index With Stroke and All‐Cause Mortality in Patients With Coronary Artery Disease: A Nationwide Cohort Study From 1999 to 2018
Source: MedComm (2020). 2025 Oct 9;6(10):e70422. doi: 10.1002/mco2.70422 (PMC12508619; doi:10.1002/mco2.70422)
Supplement: Supplementary file 1 — Supporting File 1: mco270422‐sup‐0001‐SuppMat.docx. [file MCO2-6-e70422-s001.docx]

**Association of the triglyceride-glucose index with stroke and all-cause mortality in patients with coronary artery disease: A nationwide cohort study from 1999 to 2018**

Li-Xin Huang^1#^, Tao Sun^1#^, Jun Sun^1#^, Zhi-Min Wu^1#^, Yi-Bo Zhao^1^, Ming-Yang Li^1^, Qing-Yi Huo^1^, Bao-Yu Zhang^1^, Cong Ling^1*^, Chuan Chen^1*^, Hui Wang^1*^

1 Department of Neurosurgery, The Third Affiliated Hospital of Sun Yat-Sen University, Guangzhou, China.

#These authors made equal contributions and are joint first authors.

*Hui Wang, Chuan Chen, and Cong Ling are co-corresponding authors.

*Correspondence:

Hui Wang, Chuan Chen, and Cong Ling, Department of Neurosurgery, Third Affiliated Hospital of Sun Yat-sen University, 600 Tianhe Road, Guangzhou, Guangdong 510630, China.
Email: wangh22@mail.sysu.edu.cn; chenchn6@mail.sysu.edu.cn; doctor200@163.com

| **Table S1**. Baseline characteristics of the study participants grouped by stroke status after excluding participants with missing data. | | | | |
| --- | --- | --- | --- | --- |
|  | Total (n=585) | Stroke (n=95) | Non-stroke (n=490) | P value |
| Age, years, mean (SD) | 68.6 ± 10.8 | 70.4 ± 10.7 | 68.3 ± 10.8 | 0.089 |
| Gender, n (%) |  |  |  | 0.018 |
| Male | 405 (69.2%) | 56 (58.9%) | 349 (71.2%) |  |
| Female | 180 (30.8%) | 39 (41.1%) | 141 (28.8%) |  |
| BMI, kg/m2, mean (SD) | 29.5 ± 6.1 | 29.2 ± 5.9 | 29.6 ± 6.1 | 0.582 |
| Waist circumference (cm), mean (SD) | 102.2 ± 21.3 | 99.0 ± 22.7 | 102.8 ± 21.0 | 0.113 |
| Race, n (%) |  |  |  | 0.757 |
| Hispanic | 92 (15.7%) | 16 (16.8%) | 76 (15.5%) |  |
| Non-Hispanic White | 384 (65.6%) | 59 (62.1%) | 325 (66.3%) |  |
| Non-Hispanic Black | 69 (11.8%) | 14 (14.7%) | 55 (11.2%) |  |
| Multiracial/other | 40 (6.8%) | 6 (6.3%) | 34 (6.9%) |  |
| PIR, mean (SD) | 2.5 ± 1.6 | 2.2 ± 1.4 | 2.6 ± 1.6 | 0.006 |
| Education level, n (%) |  |  |  | 0.007 |
| Below high school | 183 (31.3%) | 42 (44.2%) | 141 (28.8%) |  |
| High school graduate or GED | 132 (22.6%) | 21 (22.1%) | 111 (22.7%) |  |
| Some college or above | 270 (46.2%) | 32 (33.7%) | 238 (48.6%) |  |
| Marital status, n (%) |  |  |  | 0.318 |
| Married or living with a partner | 384 (65.6%) | 56 (58.9%) | 328 (66.9%) |  |
| Never married | 24 (4.1%) | 5 (5.3%) | 19 (3.9%) |  |
| Widowed, divorced, or separated | 177 (30.3%) | 34 (35.8%) | 143 (29.2%) |  |
| Nicotine exposure, n (%) |  |  |  | 0.802 |
| Never | 92 (15.7%) | 13 (13.7%) | 79 (16.1%) |  |
| Former | 10 (1.7%) | 2 (2.1%) | 8 (1.6%) |  |
| Now | 483 (82.3%) | 80 (84.2%) | 403 (82.2%) |  |
| Alcohol use, n (%) |  |  |  | 0.606 |
| Non-drinker | 204 (34.9%) | 37 (38.9%) | 167 (34.1%) |  |
| 1-5 drinks/month | 370 (63.4%) | 55 (58.9%) | 314 (64.3%) |  |
| 6-10 drinks/month | 2 (0.3%) | 1 (1.1%) | 1 (0.2%) |  |
| > 10 drinks/month | 3 (0.4%) | 1 (0.7%) | 2 (0.3%) |  |
| Medical history, n (%) |  |  |  |  |
| Hypertension | 531 (90.8%) | 82 (86.3%) | 449 (91.6%) | 0.101 |
| Diabetes | 233 (39.8%) | 44 (46.3%) | 189 (38.6%) | 0.158 |
| Heart failure | 163 (27.9%) | 46 (43.2%) | 122 (24.9%) | 0.001 |
| Angina/angina pectoris | 194 (33.2%) | 36 (37.9%) | 158 (32.2%) | 0.284 |
| Cancer | 123 (21.0%) | 26 (27.4%) | 97 (19.8%) | 0.128 |
| Antihyperlipidemic | 427 (73.0%) | 68 (71.6%) | 359 (73.3%) | 0.735 |
| Antidiabetic | 161 (27.5%) | 27 (28.4%) | 134 (27.3%) | 0.830 |
| SBP, mmHg, mean (SD) | 132.5 ± 21.7 | 132.7 ± 24.4 | 132.4 ± 21.2 | 0.912 |
| DBP, mmHg, mean (SD) | 66.6 ± 12.7 | 65.6 ± 13.1 | 66.8 ± 12.6 | 0.373 |
| Laboratory measurements, mean (SD) |  |  |  |  |
| HbA1c, % | 6.23 ± 1.27 | 6.23 ± 1.38 | 6.23 ± 1.24 | 0.927 |
| FBI, pmol/L | 89.8 ± 12.9 | 95.4 ± 17.3 | 88.8 ± 11.6 | 0.001 |
| TC, mmol/L | 4.42 ± 1.12 | 4.48 ± 1.44 | 4.41 ± 1.16 | 0.631 |
| HDL, mmol/L | 1.29 ± 0.40 | 1.33 ± 0.46 | 1.28 ± 0.39 | 0.288 |
| LDL, mmol/L | 2.45 ± 0.96 | 2.52 ± 1.20 | 2.44 ± 0.91 | 0.452 |
| ALB, g/L | 41.5 ± 3.3 | 40.7 ± 3.5 | 41.7 ± 3.3 | 0.009 |
| ALT, IU/L | 23.7 ± 14.8 | 22.4 ± 14.0 | 23.9 ± 14.9 | 0.370 |
| AST, IU/L | 25.4 ± 10.4 | 25.3 ± 10.2 | 25.4 ± 10.0 | 0.943 |
| BUN, mmol/L | 6.58 ± 3.37 | 7.10 ± 3.66 | 6.48 ± 3.30 | 0.101 |
| GGT, IU/L | 33.6 ± 40.7 | 31.2 ± 33.3 | 34.0 ± 42.0 | 0.526 |
| LDH, IU/L | 146.4 ± 36.2 | 151.0 ± 33.5 | 145.5 ± 36.7 | 0.174 |
| TBIL, umol/L | 12.8 ± 6.0 | 12.1 ± 6.0 | 13.0 ± 6.0 | 0.202 |
| SUA, IU/L | 365.1 ± 94.7 | 358.0 ± 99.0 | 366.5 ± 93.9 | 0.423 |
| SCR, umol/L | 100.9 ± 69.8 | 108.4 ± 64.0 | 99.5 ± 70.8 | 0.257 |
| TG, mg/dL | 137.0 ± 84.4 | 168.6 ± 117.9 | 130.9 ± 74.9 | < 0.001 |
| FBG, mg/dL | 121.6 ± 41.5 | 136.8 ± 64.1 | 118.6 ± 34.8 | < 0.001 |
| TyG index, mean (SD) | 8.86 ± 0.68 | 9.04 ± 0.89 | 8.83 ± 0.62 | 0.001 |
| All-cause mortality, n (%) | 222 (37.9%) | 46 (48.4%) | 176 (35.9%) | 0.022 |
| Cardiovascular mortality, n (%) | 17 (7.3%) | 14 (14.7%) | 3 (0.6%) | < 0.001 |
